# Semi- and fully synthetic carbohydrate vaccines against pathogenic bacteria: recent developments

**DOI:** 10.1042/BST20210766

**Published:** 2021-09-08

**Authors:** Magdalena E. Zasłona, A. Michael Downey, Peter H. Seeberger, Oren Moscovitz

**Affiliations:** 1 Department of Biomolecular Systems, Max Planck Institute of Colloids and Interfaces, Potsdam, Germany; 2 Institute of Chemistry and Biochemistry, Freie Universität Berlin, Berlin, Germany

**Keywords:** antibacterial vaccines, bacteria, capsular polysaccharides, carbohydrates, synthetic carbohydrate, vaccines

## Abstract

The importance of vaccine-induced protection was repeatedly demonstrated over the last three decades and emphasized during the recent COVID-19 pandemic as the safest and most effective way of preventing infectious diseases. Vaccines have controlled, and in some cases, eradicated global viral and bacterial infections with high efficiency and at a relatively low cost. Carbohydrates form the capsular sugar coat that surrounds the outer surface of human pathogenic bacteria. Specific surface-exposed bacterial carbohydrates serve as potent vaccine targets that broadened our toolbox against bacterial infections. Since first approved for commercial use, antibacterial carbohydrate-based vaccines mostly rely on inherently complex and heterogenous naturally derived polysaccharides, challenging to obtain in a pure, safe, and cost-effective manner. The introduction of synthetic fragments identical with bacterial capsular polysaccharides provided well-defined and homogenous structures that resolved many challenges of purified polysaccharides. The success of semisynthetic glycoconjugate vaccines against bacterial infections, now in different phases of clinical trials, opened up new possibilities and encouraged further development towards fully synthetic antibacterial vaccine solutions. In this mini-review, we describe the recent achievements in semi- and fully synthetic carbohydrate vaccines against a range of human pathogenic bacteria, focusing on preclinical and clinical studies.

## Introduction

Over their 200-year history, vaccines have become the most cost-effective medical intervention to prevent morbidity and mortality worldwide. Mainly due to developments of the last 50 years, vaccines have improved human health enormously. Vaccines have controlled and, in some cases, eradicated many viral (smallpox, measles, polio) and bacterial (diphtheria, tetanus) infectious diseases.

Carbohydrates, present on the outermost surface of all cells, can serve as potent antigenic targets for vaccine design much like protein antigens. Moreover, carbohydrate-based vaccines can broaden the molecular palette of potential targets to combat a wide range of pathogens [1]. Traditionally, carbohydrate-based vaccines have resulted from purified carbohydrate antigens from cultivated pathogens; however, carbohydrate-based vaccines isolated from pathogen cultures are of heterogeneous composition, may contain impurities, and necessitate a complicated manufacturing process.

These problems have been solved by the introduction of synthetic carbohydrate-based vaccines. Since the introduction of the first commercialized synthetic carbohydrate-based vaccine Quimi-Hib (CIGB, Cuba) to the market in 2004 [2], this field has burgeoned. Improvements in the chemical synthesis of carbohydrates, especially due to the introduction of automated glycan assembly, have facilitated access to these crucial synthetic carbohydrate epitopes.

This mini-review describes the most significant developments in fully and semisynthetic antibacterial carbohydrate-based vaccines since 2016. We focus on preclinical and clinical studies of vaccines targeting *Streptococcus pneumoniae*, *Streptococcus* Groups A and B, *Neisseria meningitis*, *Shigella flexneri*, *Salmonella enterica*, *Vibrio cholerae*, and *Burkholderia pseudomallei* and *mallei*. We refer the interested reader to a recent comprehensive review that provides an in-depth examination of synthetic-carbohydrate technology and its use against bacterial infections [3].

## The composition of carbohydrate-based vaccines: carrier proteins and adjuvants

**Figure 1.**
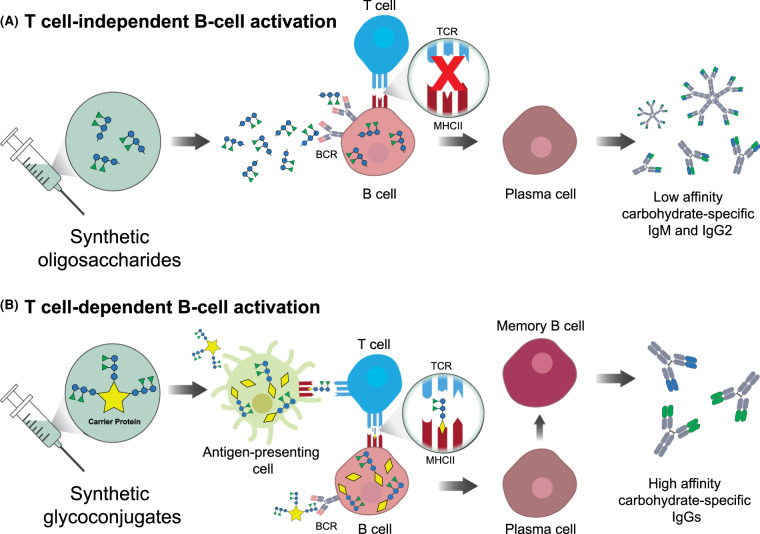
T cells-dependent and independent activation of B cells by synthetic oligosaccharides. (**A**) Synthetic oligosaccharides bind B-cell receptors (BCRs) but cannot be presented by the MHCII complexes to T-cell receptors (TCRs). B cell uptake of oligosaccharides launches plasma cells development and low-affinity antibody production. (**B**) Synthetic glycoconjugates are composed of oligosaccharides coupled with a carrier protein. Glycoconjugates internalization and processing by antigen-presenting cells (APCs) enables the presentation of glycopeptides on the MHCII complexes. MHCII binding and activation of TCRs result in memory B cells formation and high affinity IgGs development.

The main obstacle in the design of carbohydrate-based vaccines is the poor immunogenicity of pure carbohydrate antigens in high-risk groups, such as infants and the elderly [1]. Carbohydrate-based vaccines against infectious diseases are immunogenic and protective in older children and adults; however, stimulation of the immune system triggered by glycans is not efficient in the first years of life [4–6]. Carbohydrate antigens do not induce T-cell-dependent immunological responses, which results in a lack of immunological memory to the pathogens. The primary immune response to carbohydrate antigens results in the production of low-affinity and short-lived immunoglobulin (Ig) M antibodies. Moreover, repeated exposure to the antigen does not improve the immunological response. The finding by Avery and Goebel almost a century ago that immunogenicity of polysaccharides is enhanced by coupling to a carrier protein was an essential milestone in vaccine history that led eventually to today's commercial glycoconjugate vaccines [7]. Glycoconjugate vaccines contain carbohydrate antigens conjugated to a carrier protein. In contrast with non-conjugated carbohydrate antigens, glycoconjugates are processed by antigen-presenting cells and presented by the major histocompatibility complex (MHC) class II molecules to T cells. MHC-II presentation then activates both B- and T-cell immune responses that lead to IgM-to-IgG class switching, differentiation of glycan-specific memory B cells, and long-lived T-cell memory [8,9] (Figure 1).

Pioneering work by the Robbins and Schneerson team at NIH led to the first polysaccharide conjugate vaccine against *Haemophilus influenzae* type b (Hib) [10] composed of a carbohydrate moiety conjugated to a carrier protein, licensed between 1987 and 1990 [11]. There are currently five carrier proteins used in licensed conjugate vaccines and shown in clinical trials to increase glycoconjugate vaccines immunogenicity: (1) diphtheria toxoid (DT); (2) the genetically modified cross-reacting material of diphtheria toxin (CRM)_197_; (3) tetanus toxoid (TT); (4) outer membrane protein complex (OMPC) from serogroup B meningococcus; (5) and *Haemophilus influenzae* protein D (HiD). The main differences between the carrier proteins are the maximal number of conjugated carbohydrates and the type of antibodies and immune response they elicit (for a recent comprehensive review, see [11].

Some vaccines either in the developmental phase or already licensed may additionally contain adjuvants. Adjuvants increase the uptake of target antigens, including carbohydrate antigens, which results in better immunogenicity of the vaccine formulation. Many external adjuvants have been developed to augment the immunogenicity of the experimental and clinical vaccines, including mineral salts, liposome emulsions, inactivated bacteria, protein toxins, immune cell receptors activating ligands, and aluminium salts as adjuvants [12].

## Traditional carbohydrate-based vaccines

Most carbohydrate-based vaccines consist of native carbohydrate antigens that induce an effective immune response against the respective pathogens. Despite their tremendous efficacy, native carbohydrate vaccines face several drawbacks. First, the inherent structural heterogeneity of native carbohydrates may result in batch-to-batch variation and efficiency of the glycoconjugate vaccines. Cell impurities, such as proteins and nucleic acids, may pose a risk when used for immunization. As a result, purifying native carbohydrates involves complicated, expensive, and time-consuming steps required for a successful and safe vaccine manufacturing process [13,14]. Culturing large numbers of pathogens is another limiting factor with safety concerns for personnel and the environment. Different pathogens cannot be cultivated at a sufficient scale, or it is impossible to cultivate them in a scalable setting for manufacturing purposes. The presence of several functional groups with the potential to generate a link with the carrier protein may result in uncontrolled and unreproducible conjugation that may alter the specificity, level, and type of the desired immune response [15]. Thus, vaccines based on cultivated pathogens require high-quality standards for formulations, which relates to difficulties in obtaining regulatory approvals [16]. All carbohydrate-based vaccines currently approved by the FDA, which are exclusively traditionally obtained, are listed in Table 1.

**Table 1:** Carbohydrate-based vaccines approved by the FDA

Antigen	Indications	Trade name	Manufacturer	Carrier protein	Adjuvant	Age approval age
*Haemophilus influenzae* type b; CPS (polyribosyl-ribitol-phosphate)	invasive disease caused by *Heamophilus influenzae* type b	HIBERIX	GlaxoSmithKline Biologicals	TT	-	children 6 weeks–4 years
		ActHIB	Sanofi Pasteur	TT	-	children 2 months–5 years
		Liquid PedvaxHIB	Merck Sharp & Dohme	OMPC	amorphous aluminium hydroxyphosphate sulfate	children 2 months–5 years
	diphtheria, tetanus, pertussis, poliomyelitis, and invasive disease caused by *Heamophilus influenzae* type b	Pentacel	Sanofi Pasteur	TT	aluminium phosphate	children 6 weeks–4 years
	diphtheria, tetanus, pertussis, poliomyelitis, hepatitis B, and invasive disease caused by *Heamophilus influenzae* type b	VAXELIS	MCM Vaccine	OMPC	aluminium salts (various)	children 6 weeks–4 years
*Neisseria meningitidis* serogroups A, C, Y and W-135 (Menactra, Menveo, Menomune-A/C/Y/W-135) or serogroup W (MenQuadfi); CPS	invasive meningococcal disease caused by *Neisseria meningitidis* serogroups A, C, Y, and W-135 or W	Menactra	Sanofi Pasteur	DT	-	9 months–55 years
		MENVEO	GlaxoSmithKline Biologicals SA	CRM_197_	-	2 months–55 years
		Menomune-A/C/Y/W-135	Sanofi Pasteur	-	-	≥2 years
		MenQuadfi	Sanofi Pasteur	TT	-	≥2 years
*Salmonella enterica* serovar Typhi; cell surface Vi polysaccharide	typhoid fever caused by *Salmonella enterica* serovar Typhi	Typhim Vi	Sanofi Pasteur	-	-	≥2 years
*Streptococcus pneumoniae* serotypes 1, 3, 4, 5, 6A, 6B, 7F, 9V, 14, 18C, 19A, 19F, and 23F; CPS	invasive disease caused by *Streptococcus pneumoniae* serotypes 1, 3, 4, 5, 6A, 6B, 7F, 9V, 14, 18C, 19A, 19F, and 23F	Prevnar 13	Wyeth Pharmaceuticals	CRM_197_	aluminium phosphate	children 6 weeks-5 years; children 6 years-17 years; adults ≥18 years
*Streptococcus pneumoniae* serotypes 1, 2, 3, 4, 5, 6B, 7F, 8, 9N, 9V, 10A, 11A, 12F, 14, 15B, 17F, 18C, 19F, 19A, 20, 22F, 23F, and 33F; CPS	invasive disease caused by *Streptococcus pneumoniae* serotypes 1, 2, 3, 4, 5, 6B, 7F, 8, 9N, 9V, 10A, 11A, 12F, 14, 15B, 17F, 18C, 19F, 19A, 20, 22F, 23F, and 33F	PNEUMOVAX 23	Merck & Co.	-	-	≥50 years and persons ≥2 years who are at increased risk of pneumococcal disease

Currently, all the approved vaccines are traditionally obtained, consisting of carbohydrate antigens purified from isolated antigens.

This table was adopted from the complete list of ‘Vaccines Licensed for Use in the United States' provided by the US Food and Drug Administration website (https://www.fda.gov/). CPS: capsular polysaccharide.

Content current as of: March 8, 2021.

### Synthetic carbohydrate-based vaccines

In contrast with traditional carbohydrate-based vaccines, synthetic carbohydrate formulations are highly reproducible with homogenous, well-defined composition. Their highly defined molecular carbohydrate structure may potentially lower the costs of the vaccine design and eliminate the costly cold chain, which is especially challenging to maintain in hot developing countries [13,17]. Synthetic carbohydrate-based vaccines can be divided into two categories: semisynthetic and fully synthetic.

#### Semisynthetic carbohydrate-based vaccines

**Figure 2.**
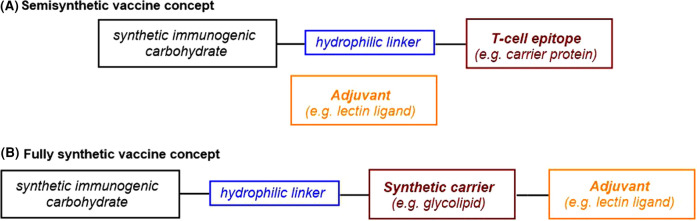
Synthetic carbohydrate-based vaccines. (**A**) Semisynthetic and (**B**) fully synthetic vaccine strategies.

Semisynthetic carbohydrate-based vaccines are comprised of mono- or multivalent glycans conjugated to a carrier protein, and an adjuvant is co-administered as a separate molecule (Figure 2A) [3,18]. One of the most commonly used carrier proteins is CRM_197._ Although semisynthetic vaccines comprised of a conventional peptide/protein carrier are still under development, the carbohydrate moiety in emerging semisynthetic vaccine candidates can be conjugated to novel carriers, for example, virus-like particles such as bacteriophage Qβ.

Superficially, the selection of the chain of atoms connecting the immunogenic glycan to the carrier protein may seem trivial; however, to synthetic chemists, this ‘linker' is especially important: it must usually contain hydrophilic scaffolding, such as ether and/or amide functional groups, to ensure solubility while surviving multiple synthetic steps (Figure 2A).

#### Fully synthetic carbohydrate-based vaccines

In fully synthetic carbohydrate-based vaccines, the carbohydrate moiety conjugated to the synthetic carrier, such as a glycolipid, synthetic peptide, or a nanoparticle, is combined with different adjuvants all to form one (macro)molecule (Figure 2B). Interestingly, even in the absence of a carrier protein, fully synthetic solutions based on glycolipids carrying liposomes, zwitterionic polysaccharides, or carbohydrate-based nanoparticles induce T cell-dependent response through different mechanisms successfully [3,18,19]. Although co-localization of the elements in the same molecule triggers a more potent antibody response than in the case of semisynthetic vaccines, none of the fully synthetic vaccine formulations is currently marketed [20]. Since 2016, only one self-adjuvating fully synthetic vaccine for *Neisseria meningitis* Group C includes the glycolipid monophosphoryl lipid A (MPLA), a modified form of lipid A, the lipophilic constituent of bacterial lipopolysaccharides [21].

We turn our attention now to the strides made in synthetic antibacterial vaccines.

## Antibacterial semisynthetic carbohydrate-based vaccines

To make polysaccharide vaccines more reproducible and cost effective, the nature-derived bacterial polysaccharides are replaced with synthetic structures to make semisynthetic vaccines. Below, we describe semisynthetic and one fully synthetic vaccine candidates in the discovery phase and in clinical trials aimed at improving currently existing antibacterial vaccines or at elucidating new vaccine candidates for combating bacterial infections. All semi- and fully synthetic antibacterial glycoconjugate vaccine candidates described in this mini-review are divided based on the bacteria they target and listed in Table 2 and Table 3 (Gram-positive and Gram-negative bacteria, respectively).

**Table 2:** Semisynthetic glycoconjugate vaccine candidates of selected Gram-positive bacteria

Bacterium and serotype (ST)	Type of glycan	Saccharide (identified as most promising)	Approach	Carrier protein	Preclinical evaluation	Ref.
*S. pneumoniae* pentavalent semisynthetic glycoconjugate vaccine ST2, ST3, ST5, ST8, ST14	CPS	oligosaccharides depending on the STs	Semi-synthetic	CRM_197_	rabbits	[35]
*S. pneumoniae* ST1		trisaccharide		CRM_197_	rabbits	[36]
*S. pneumoniae* ST2		hexasaccharide		CRM_197_	mice	[26]
*S. pneumoniae* ST3		tetrasaccharide		CRM_197_	mice	[27]
		tetrasaccharide		BSA	mice & rabbits	[37]
		hexasacchride		TT	mice	[40]
					rabbit	[41]
*S. pneumoniae* ST3 and ST14		tetrasaccharides		bacteriophage Qβ	mice	[48]
*S. pneumoniae* ST4		tetrasaccharide		CRM_197_	mice	[28]
*S. pneumoniae* ST5		oligosaccharides		CRM_197_	rabbits	[34]
*S. pneumoniae* ST8		tetrasaccharide		CRM_197_	rabbits	[30]
*S. pneumoniae* ST14		hexasaccharide		BSA	mice	[42]
		hexasaccharide		BSA	mice	[38,39]
		tetrasaccharide		pneumococcal surface adhesin A	mice	[45]
		repeating unit connected with aliphatic spacer		CRM_197_	mice	[47]
*S. pneumoniae* ST19A and ST19F		chimeric antigen comprised of a repeating unit of ST19A and ST19F CPS each		CRM_197_	rabbits	[44]
GAS various serotypes	cell-wall polysaccharide	branched oligosaccharides containing one, two and three repeating units of CPS		inactive mutant of group A streptococcal C5a peptidase (ScpA), ScpA193	mice	[50,51]
GAS various serotypes		oligorhamnoside fragments		gold nanoparticles	competitive ELISA	[52]
GBS type Ia	CPS	a dimer composed of two pentasaccharides and its corresponding monomer		CRM_197_	mice	[53]
GBS type III		hexasaccharide		CRM_197_	competitive ELISA & mice	[54–56]

**Table 3:** Semisynthetic and fully synthetic glycoconjugate vaccine candidates against selected Gram-negative bacteria

Bacteria and serotype	Type of glycan	Saccharide	Approach	Carrier protein or (molecule)	(Pre)clinical evaluation	Ref.
*N. meningitidis* serogroup A	CPS	oligosaccharide	Semi-synthetic	TT	ELISA	[65]
*N. meningitidis* serogroup A		oligosaccharide		CRM_197_	mice	[64]
*N. meningitidis* serogroup C		oligosaccharides and glycolipids	fully synthetic, self- adjuvanting	(MPLA)	mice	[21]
*N. meningitidis* serogroup C		oligosaccharide	Semi-synthetic	TT	mice	[67]
*N. meningitidis* serogroup X		oligosaccharide		CRM_197_	mice	[63]
*N. meningitidis* various strains and other pathogenic bacteria	LPS	oligosaccharide		DT	rabbits	[68]
*S. flexneri* 2a	O-polysaccharide of LPS	oligosaccharide		TT	mice	[70]
*S. flexneri* 2a		oligosaccharide		TT	Clinical trial, Phase 1, Phase 2 currently recruiting	[71]
*S.* Typhi	Vi CPS	high molecular weight polysaccharide	only polysaccharide antigen	-	molecular modeling	[74]
*S.* Enteritidis	O-polysaccharide of LPS	oligosaccharide	Semi-synthetic	bacteriophage Qβ	mice	[78]
*S.* Paratyphi A		oligosaccharide		bacteriophage Qβ	mice	[79]
*V. cholerae* O139		oligosaccharide		BSA	none	[84]
*V. cholerae* O1 serotype Inaba		glycoclusters displaying oligosaccharides		BSA	ELISA	[85]
*B. pseudomallei*	CPS	oligosaccharide		Nontoxic H_c_ domain of TT	mice	[87]
*B. pseudomallei* and *mallei*	O-polysaccharide of LPS	disaccharide		CRM_197_	mice	[88]
*B. pseudomallei* and *mallei*	CPS	oligosaccharide		CRM_197_	meliodosis patient sera	[89]

### Streptococcus pneumoniae

Infectious diseases caused by the bacterium *Streptococcus pneumoniae (S. pneumoniae)*, like pneumonia, septicemia, meningitis, and otitis media, are a global burden to medical systems and can be prevented by vaccination. Currently, over 90 serotypes (ST) of *S. pneumoniae* have been identified [22]. There are currently three licensed pneumococcal conjugate vaccines: (1) Synflorix (GlaxoSmithKline Biologicals S.A., Belgium), a decavalent vaccine distributed in Europe [23]; (2) Prevnar 13 (Wyeth Pharmaceuticals, Inc., PA, U.S.A.), a 13-valent vaccine distributed in Europe and the United States [24]; (3) and a new decavalent pneumococcal conjugate vaccine, Pneumosil (Serum Institute of India, Ltd., India) [25] launched in India at the end of 2020^1^
^1^https://www.seruminstitute.com/news_pneumosil_281220.php. Conjugates of nature-derived CPSs connected with a number of production and safety difficulties can be exchanged by semisynthetic glycoconjugate compounds to improve efficiency and coverage of pneumococcal conjugate vaccines.

**Figure 3.**
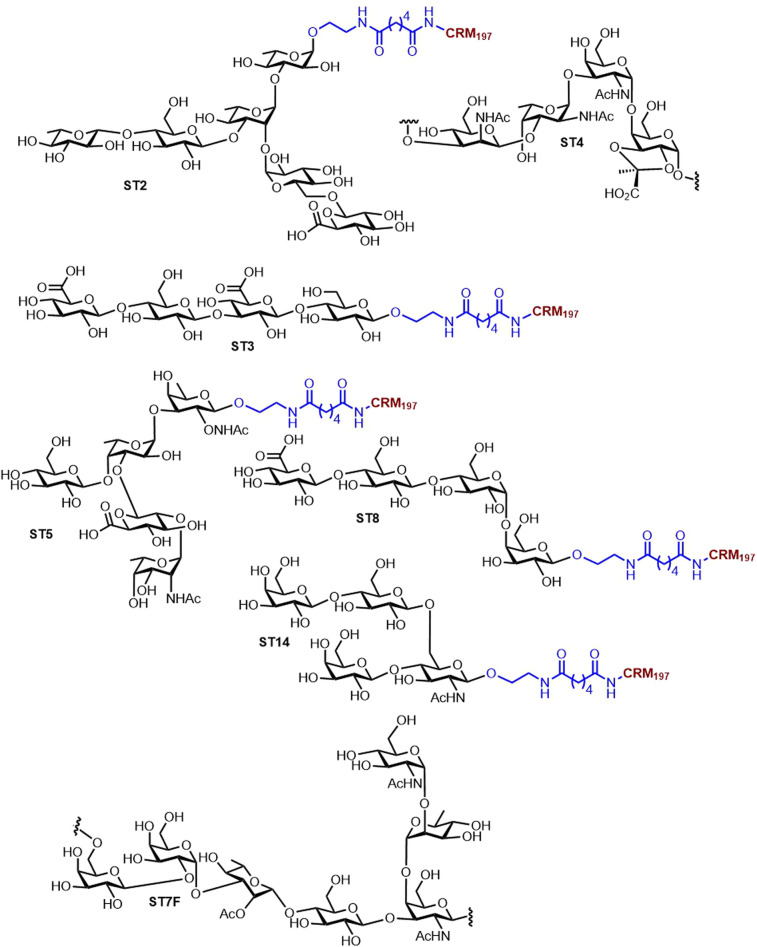
The structures of the immunogenic glycotopes in *S. pneumoniae* complete with a linker conjugated to CRM_197_. Linkers, T-cell epitopes, and adjuvant are designated in blue, maroon, and orange, respectively, throughout this survey.

We have worked toward incorporating new synthetic carbohydrates into existing pneumococcal conjugate vaccines and identifying protective and/or minimal glycan epitopes (glycotopes) of currently licensed vaccine polysaccharides. Since 2016, we have identified immunogenic and protective glycotopes of five STs: (1) ST2 (26), (2) ST3 [27], (3) ST4 [28], (4) ST7F [29], and (5) ST8 [30] (Figure 2). In all cases, glycan arrays containing synthetic repeating-unit oligosaccharides from each respective ST were used to screen reference sera for antibodies. The selected synthetic oligosaccharides of ST2, ST3, ST4, and ST8 were conjugated to CRM_197_ for in-depth immunological evaluation in animal models revealing that studies involving ST2 [26] and ST3 [27] elicited protective antibacterial immunity in mice; and ST8 [30] in rabbits. The glycoconjugate candidate of ST4 [28] was immunogenic in mice, but it had limited binding to the CPS from the respective serotype. Further analysis revealed the importance of terminal sugars in glycoconjugates for a successful immune response. Thus, the laboratory has proposed semisynthetic vaccine candidates containing relatively short synthetic oligosaccharides that may serve as promising alternatives to larger polysaccharide antigens from natural sources (Figure 3). Except for the above mentioned semisynthetic vaccine candidates, we published several syntheses of pneumococcal oligosaccharide antigens including: (1) ST9 V for better understanding of the importance of glycan modifications and further immunological evaluation [31]; (2) the total synthesis of the ST12F hexasaccharide using a new linear approach [32]; and (3) the first iterative automated glycan assembly of conjugation-ready ST3 capsular trisaccharide [33]. A glycoconjugate containing stable synthetic oligosaccharide analogs of the ST5 CPS repeating unit prevents stability issues associated with the native CPSs that include rare carbohydrates [34].

**Figure 4.**
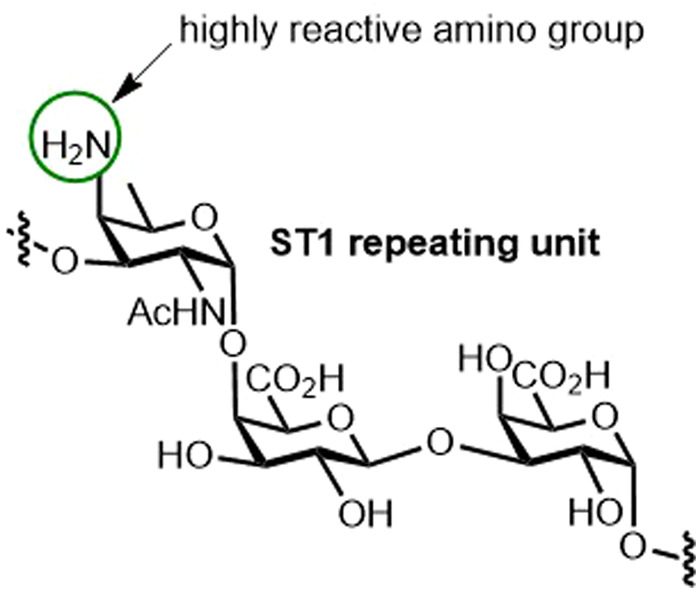
The structure of Streptococcus. pneumoniae glycotope D-AAT. Capsular ST1 bearing the crucial, yet highly reactive, D-AAT [36].

Aiming to improve Synflorix and Prevnar 13 by synthetic glycoconjugates, we proposed a pentavalent semisynthetic glycoconjugate pneumococcal vaccine that includes the following STs: (1) ST2, ST8, that have not been included in currently marketed vaccines, and ST3 absent from Synflorix; (2) ST5, which suffers from low immunogenicity or production inconsistencies; and (3) ST14 that is present in both licensed vaccines—however, it is included to show that nonproblematic STs from natural sources can easily be exchanged by synthetic glycoconjugates. The proposed vaccine conjugated to CRM_197_ elicited a robust, polysaccharide-specific antibody response compared with marketed vaccines, proving that synthetic oligosaccharides can be used in coformulation with Synflorix and Prevnar [35]. Further endeavors have focused on ST1, which in Synflorix and Prevnar 13 induces low levels of functional antibodies. An unusual monosaccharide, 2-acetamido-4-amino-2,4,6-trideoxy-D-galactose (D-AAT), present in ST1 may be responsible for the reduced vaccine efficacy. Nonetheless, a semisynthetic glycoconjugate carrying D-AAT at the nonreducing end conjugated to the CRM_197_ carrier protein elicited a strong immune response in rabbits, proving the immunological importance of D-AAT (Figure 4) [36]. Thus, the feasibility of incorporating ST1 into a semisynthetic vaccine remains to be seen.

The Nifantiev laboratory conducted intensive research on semisynthetic pneumococcal glycoconjugate vaccines. A comparative immunological study of synthetic oligosaccharides of the CPS repeating units of ST3 [37] and ST14 [38,39] conjugated to bovine serum albumin (BSA) revealed that the optimal candidates for semisynthetic vaccines for both STs are tetrasaccharide ligands. Further immunological studies in mice revealed protective properties of both neoglycoconjugates. Nifantiev and coworkers compared glycotopes from di- to tetrasaccharides of ST3. Meanwhile, a comparative study of penta- to octasaccharides from the ST3 CPS concluded that a hexasaccharide-TT glycoconjugate is the most promising [40,41]. Nifantiev conducted the first study on the activation of the innate immune system in response to the semisynthetic glycoconjugate and selected a hexasaccharide of CPS ST14 conjugated to BSA as a vaccine candidate [42].

**Figure 5.**
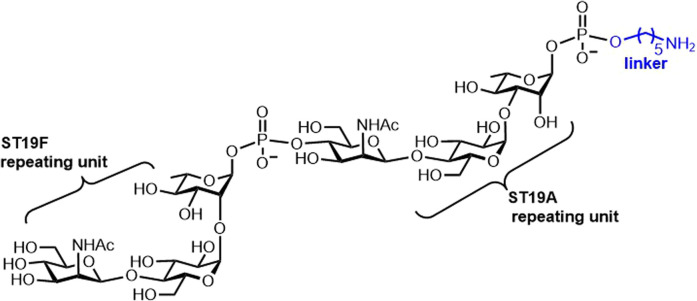
Synthetic carbohydrate antigens from Streptococcus. pneumoniae. Chimeric antigen bearing both ST19F and ST19A [44].

Recent studies concerning the *S. pneumoniae* synthetic carbohydrate-based vaccine candidates involved chemical strategies to access the repeating unit of CPS ST19A [43]. A chimeric antigen containing both repeating units of the ST19A and ST19F CPSs conferred substantial immunogenicity in rabbits. Antibodies produced in response to the semisynthetic chimeric glycoconjugate antigen neutralized ST19A and ST19F bacteria, while the conjugates containing other glycan epitopes failed (Figure 5) [44]. A glycoconjugate ST14 pneumococcal vaccine with unique carrier-pneumococcal surface adhesin A (PsaA) [45] investigated the role of the conjugation site in determining the immunogenicity of the glycoconjugate [46]. Connecting the repeating unit of ST14 to an aliphatic spacer to simplify syntheses based on glycosidic linkages between repeating units of the ST14 CP yielded a neo-glycoconjugate with CRM_197_ as a promising alternative with the advantage of a more straightforward synthesis [47].

To improve glycoconjugate vaccines in general, the relatively short linear ST3 and branched ST14 carbohydrate epitopes were attached to a virus-like particle (VLP) as a carrier to initiate both B and T cell-dependent immune responses, enabling loading of ∼20–200 antigens per VLP for both ST3 and ST14. The formulation was completed with a powerful adjuvant—an agonist glycolipid of NKT cells. This proof-of-concept glycoconjugate elicited serotype-specific, protective, and long-lasting IgG antibodies of nanomolar affinity in mice, proving the improved qualities of the new model [48].

### Streptococcus Groups A and B

The majority of Group A *Streptococcus* (GAS) infections cause relatively mild illnesses but can develop into the sometimes lethal streptococcal toxic shock syndrome. In total, due to diseases caused by GAS, over half a million people die globally each year. Since GAS is sensitive to commonly used antibiotics, constantly rising antibiotic resistance is of increasing concern regarding GAS infections and prophylaxis; thus, an effective vaccine remains a priority [49].

**Figure 6.**
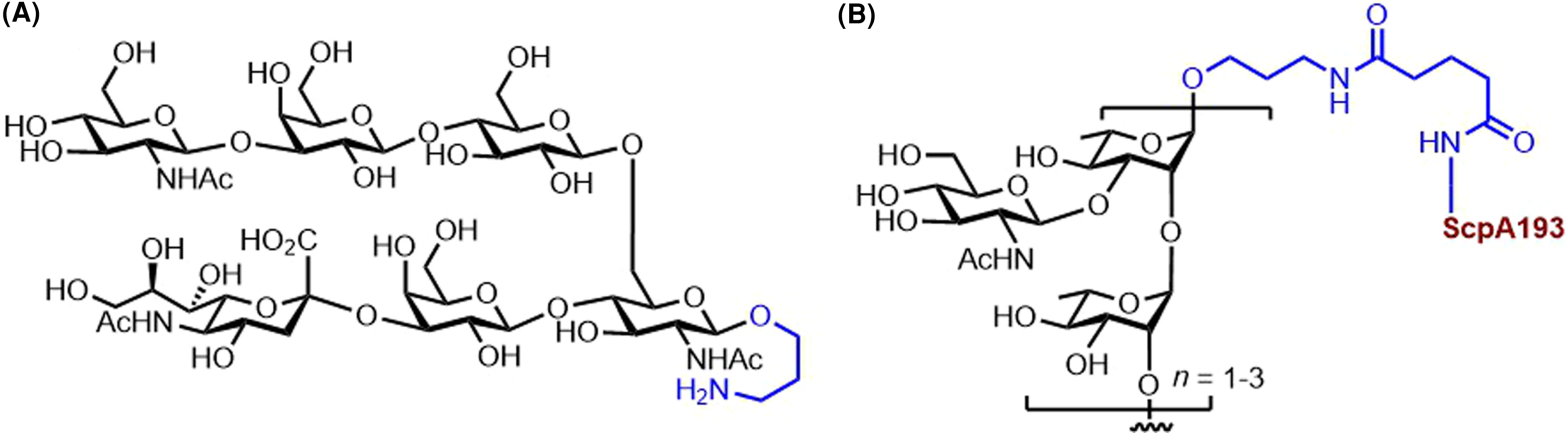
Glycotope and a semisynthetic vaccine conjugate from capsular polysaccharides of Group A and B Streptococcus. (**A**) ‘Diluted' hexasaccharide containing only the key residues necessary to elicit an immune response to Group B *Streptococcus* type III [54–56]. (**B**) Group A CPS tethered to the ScpA193 protein conferring bivalent immunity [50,51].

A bivalent CPS–ScpA193 vaccine conjugate composed of synthetic oligosaccharides from the repeating unit of the major and the conserved cell wall polysaccharide from GAS STs conjugated to ScpA193, a novel streptococcal C5a peptidase (ScpA) mutant as the protein carrier. ScpA was identified as a potential target antigen for the development of an anti-GAS vaccine due to its high immunogenicity. Novel glycoconjugates with the ScpA193 protein elicited a strong carbohydrate–antigen-specific antibody response in mice. Moreover, the ScpA193 conjugates induced a robust anti-ScpA antibody response, proving the bivalency of the CPS–ScpA193 conjugate against GAS (Figure 6B) [50]. Immunological studies in mice revealed that the nonasaccharide–ScpA193 glycoconjugate is a promising GAS vaccine candidate [51]. Recent advances in the design of synthetic GAS glycoconjugate vaccines involve gold nanoparticles as a nano-vaccine platform. The carbohydrate structure in the proposed nano-vaccine were oligorhamnoside fragments, alternative antigens for anti-GAS vaccine, common to the cell wall of all GAS strains [52].

Group B *Streptococci* (GBS) are the leading cause of bacterial sepsis and meningitis in neonates and a significant source of neonatal morbidity globally. The vaccination of mothers in the third trimester against GBS is a promising alternative to antibiotics delivered intrapartum [57]. Attempts toward a usable semisynthetic vaccine, described below, concern six out of nine GBS STs.

Since 2016, several synthetic methods for the oligosaccharide repeating units of GBS CPS were published: [1] a dimer of the branched polysaccharide from ST Ia [53]; (2) regioselective routes to building blocks that accessed the ST Ia and Ib repeating units [58]; (3) the synthesis of three different pentasaccharide frameshifts of ST III [54] that were later investigated as a decasaccharide fragment composed of two repeating units to reveal the portion necessary for antibody recognition [56]; (4) a branched heptasaccharide of the repeating unit of ST II [59]; (5) ST V [60]; and (6) a hexasaccharide repeating unit with the dimer of this repeating unit from ST VII [61]. A dimer of the branched pentasaccharide repeating unit from ST Ia and its corresponding monomer conjugated to CRM_197_ resulted in robust antibody response in mice [53]. A dimer fragment composed of two pentasaccharide repeating units from ST III could be shortened to a hexasaccharide representing the minimal antigenic portion. After subsequent conjugation to the CRM_197_ carrier protein, the semisynthetic vaccine candidate elicited immune responses at similar levels to the native polysaccharide conjugate (Figure 6A) [54–56].

### Neisseria meningitidis

*Neisseria meningitidis* (*N. meningitis*) is a Gram-negative bacterium causing bacterial meningitis and sepsis. Among the twelve different serogroups, the six most common serogroups, A, B, C, W, X, and Y, cause most of the epidemics globally. Meningococcal infections can be prevented with vaccines [62]. Similar to the vaccines preventing *S. pneumoniae* infections, there are multiple vaccines available against *N. meningitidis* based on isolated CPSs, for example, polysaccharide vaccine Menomune-A/C/Y/W-135 (Sanofi Pasteur Inc., PA, U.S.A.)^2^
^2^https://www.fda.gov/media/83562/download and MenQuadfi (Sanofi Pasteuer Inc., PA, U.S.A.)^3^
^3^https://www.fda.gov/media/137306/download, a newly US-approved conjugate vaccine. Nevertheless, to date, there is no licensed vaccine against serogroup X, and scientific efforts aim to improve licensed vaccines with semisynthetic glycoconjugates and provide vaccine solutions against the increasing rates of serogroup X are necessitated [62].

**Figure 7.**
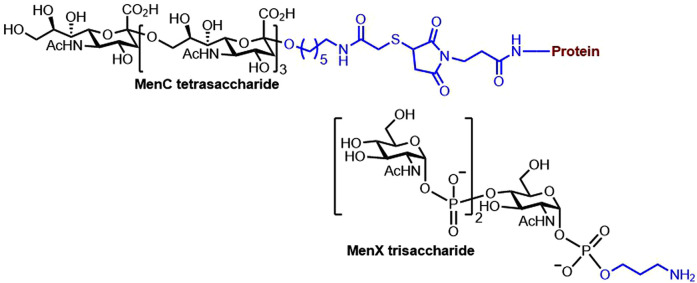
The structure of a glycotope and a glycoconjugate vaccine candidate against Neisseria meningitidis. Semisynthetic glycoconjugates of *N. meningitidis* serogroup X [63] and C [67].

A rapid and cost-effective chemoenzymatic synthesis of oligosaccharides from *N. meningitidis* serogroup X produced glycans that elicited functional antibodies in mice (Figure 7 bottom) [63]. A vaccine candidate involving glycomimetics that stabilizes the natural CPS of *N. meningitis* serogroup A after conjugation to CRM_197_ elicited in mice high levels of protective antibodies with bactericidal activity [64], which advanced previous work that had used the synthetic tetrasaccharide conjugate of TT [65]. The structural epitopes of *N. meningitidis* serogroup A revealed the importance of acetylation of the respective CPS [66]. A semisynthetic vaccine composed of oligosaccharides from the *N. meningitidis* serogroup C conjugated to the TT proved antigenic in mice. The vaccine candidate elicited similar levels of IgG antibodies to those elicited by a commercial tetravalent vaccine (Figure 7, top) [67].

**Figure 8.**
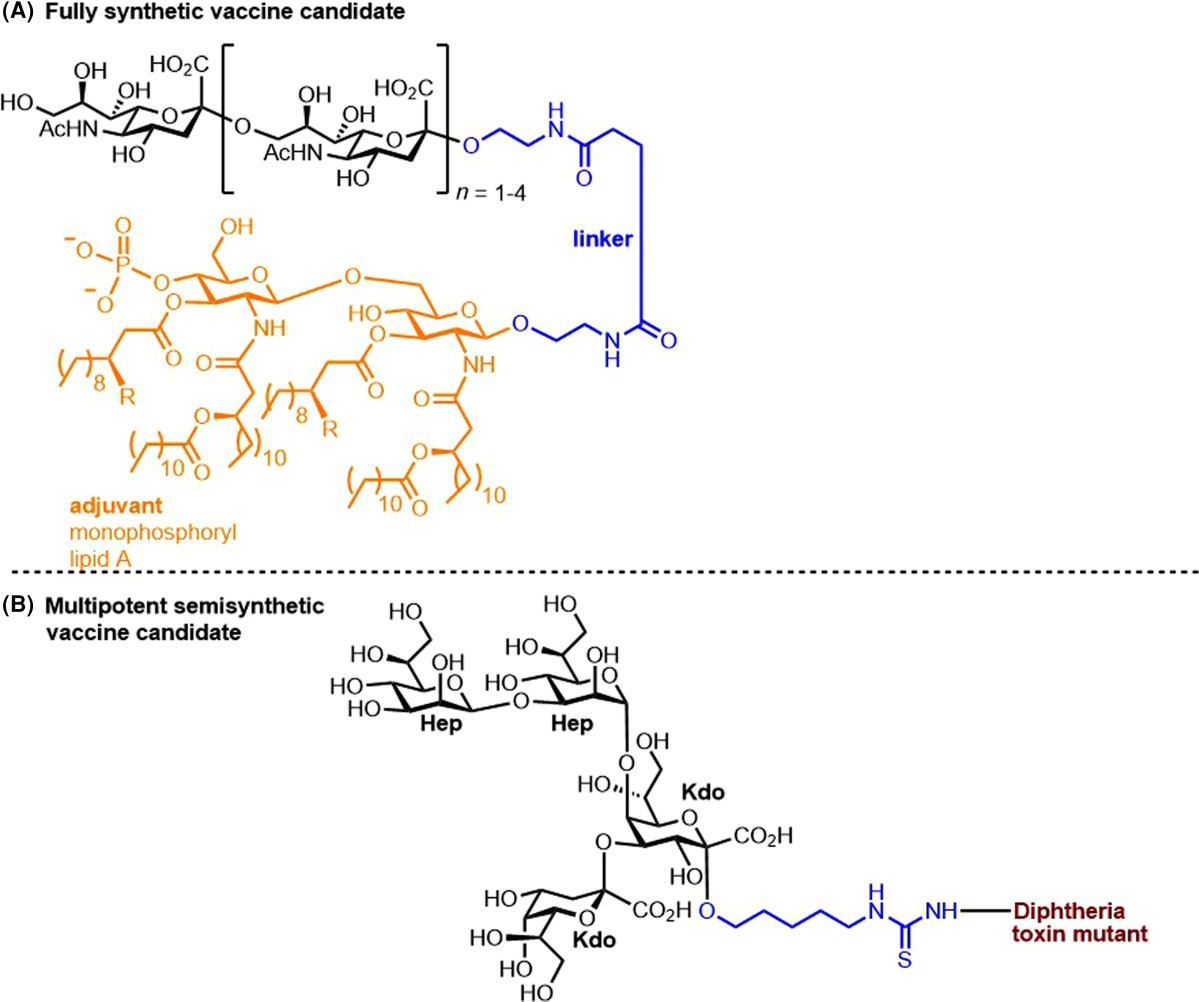
The structures of fully synthetic and multipotent vaccine candidates against Neisseria meningitidis. (**A**) Fully synthetic vaccine target against *N. meningitidis* [21]. (**B**) Semisynthetic multipotent vaccine candidate against *N*. *meningitidis* and other bacteria [68].

In addition to the many semisynthetic vaccine candidates targeting various *N. meningitidis* serogroups, the first fully synthetic vaccine candidate targeting *N. meningitidis* serogroup C is composed of oligosialic acids, an important CPS expressed by serogroup C *N. meningitidis* and the strong self-adjuvating immunostimulant MPLA glycolipid (Figure 8A). Immunological studies of this fully synthetic conjugate in mice revealed robust immune responses, proving that this vaccine candidate is worth further investigation [21]; however, to date, no clinical studies have been further reported. A core lipotetrasaccharide Hep_2_Kdo_2_, a common motif in bacterial LPS, was selected as a vaccine candidate targeting various *N. meningitidis* strains and other pathogenic bacteria. The first synthesis of this tetrasaccharide, followed by its conjugation to the DT carrier protein, yielded glycoconjugates that resulted in antibodies capable of binding not only to *N. meningitidis* strains but also *Pseudomonas aeruginosa* and *Escherichia coli* (Figure 8B) [68].

### Shigella flexneri

Shigellosis, a common debilitating diarrheal disease, especially among children under five in developing countries, is caused by Gram-negative bacteria of the genus *Shigella*. The most prevalent strain worldwide is *Shigella flexneri* (*S. flexneri*) 2a [69]. There are currently no licensed vaccines against *Shigella*.

A bioconjugation protocol was tested on a vaccine candidate for *S. flexneri 2a* infections, and it highlights the importance of the hapten:carrier ratio for immunogenicity. The semisynthetic glycoconjugate with TT was immunogenic in mice [70]. Another semisynthetic vaccine candidate, SF2a–TT15, comprised of the oligosaccharide from the repeating unit of the O antigen from *S. flexneri* 2a conjugated to the TT carrier protein has successfully been tested in a phase I clinical trial [71] and a phase 2 (NCT04078022) trial is currently recruiting [72].

### Salmonella enterica

*Salmonella enterica* serovar Typhi (*S*. Typhi), a pathogenic agent causing typhoid fever, leads to millions of infections annually worldwide. The currently licensed polysaccharide vaccine, Typhim Vi (Sanofi Pasteur SA, France), contains cell surface Vi polysaccharide from *S.* Typhi isolated from wild-type bacteria [73]. A vaccine candidate comprised of the synthetic Vi antigen (GelSite-OAc™) based on *O*-acetylation of a novel high-molecular-weight polygalacturonic acid (GelSite) from *Aloe vera* was proposed since the high-molecular-weight polygalacturonic acid shares the same backbone. Interestingly, the pure polysaccharide elicited a boosting effect and an increase in specific IgG levels, indicating a T cell-dependent response even in the absence of a carrier protein. Immunological evaluation in mice showed induction of a strong antigen-specific and protective antibody response that was comparable to or higher than the antibody response induced by the licensed Vi vaccine, proving that this polysaccharide could be effective against *S.* Typhi [74].

**Figure 9.**
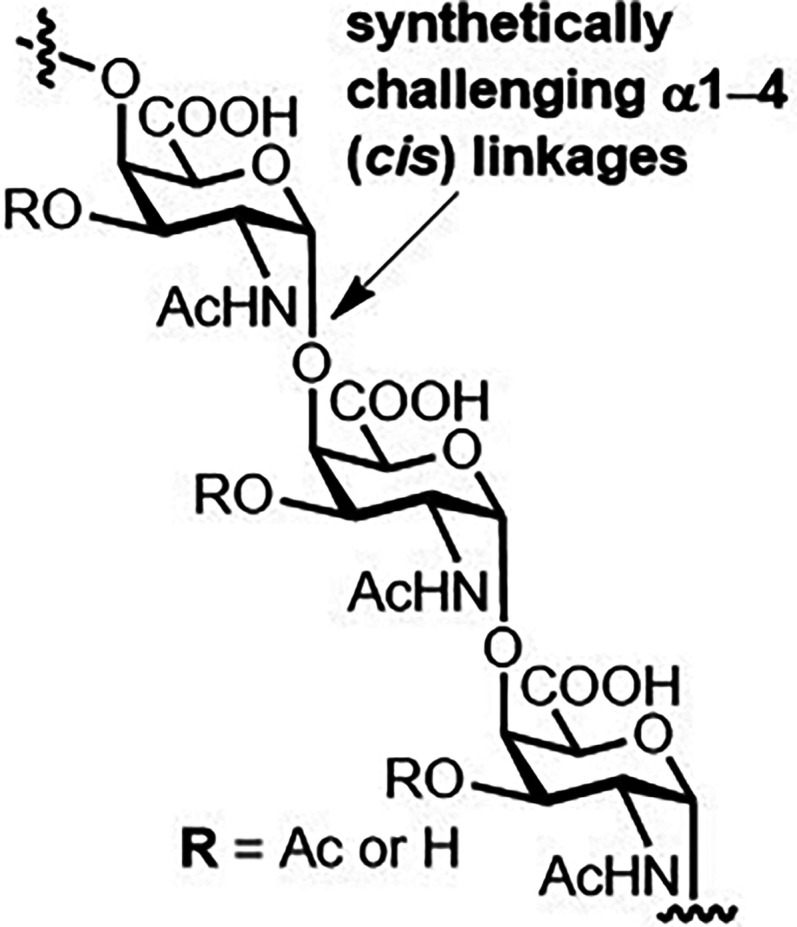
Glycotope structure from Salmonella. The Vi CPS of *S.* Typhi bearing synthetically daunting α1–4 (*cis*) glycosidic linkages [75,76].

The synthetic approach is promising but complicated due to the presence of repeating α1,4 (*cis*) linkages found in the Vi CPS of *S.* Typhi (Figure 9). After the importance of the acetyl groups in providing antigenicity [75] was realized, Vi pseudooligosaccharides mimicking the Vi polysaccharide from *S.* Typhi were synthesized, while simplifying the synthesis of the full-length oligosaccharides [76]. A series of synthetic Vi oligosaccharides revealed the hexasaccharide as the possible minimum epitope of the Vi antigen from *S*. Typhi [77].

**Figure 10.**
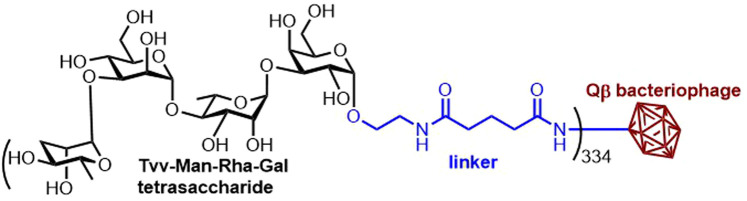
The structure of a protective Qβ-glycoconjugate against Salmonella Enteriditis. *S. Enteriditis* vaccine candidate employing the Qβ bacteriophage [78].

*Salmonella enterica* serovar Enteriditis (*S.* Enteriditis) is one of the most prevalent strains of non-typhoidal *Salmonella* and the causative agent of serious and fatal systemic infections. There are currently no licensed vaccines preventing non-typhoidal *Salmonella* infections. The first synthetic carbohydrate-based anti-*S. Enteriditis* vaccine candidate is based on conjugation of the synthetic tetrasaccharide repeating unit of the *S. Enteriditis* O-polysaccharide of LPS to the bacteriophage Qβ as the carrier. The bacteriophage Qβ was shown to be an effective carrier in anticancer and anti-inflammation vaccines. Nevertheless, this study utilizes the bacteriophage Qβ in a carbohydrate-based antibacterial vaccine for the first time. The tetrasaccharide — Qβ conjugates elicited a potent IgG antibody response in mice and rabbits (Figure 10) [78]. Later, the same concept of a vaccine candidate was tested with synthetic oligosaccharides from *Salmonella enterica* serovar *Paratyphi A* exhibiting potent IgG antibody responses in animals [79].

### Vibrio cholerae

**Figure 11.**
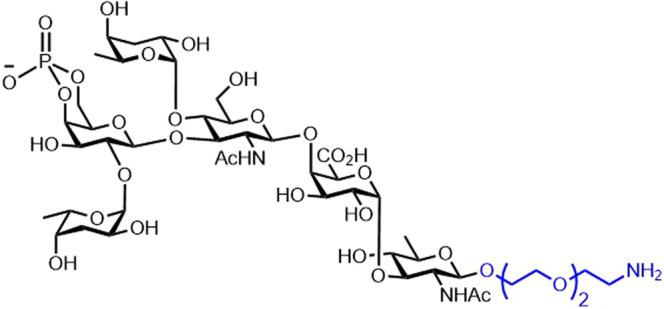
Synthetically available antigen of *V. cholerae* [80]. O-antigen

*Vibrio cholerae* (*V. cholerae)* is a pathogenic agent causing cholera, a severe diarrheal disease that can be fatal if left untreated. Synthetic LPS structures from *V. cholerae* O1, STs Ogawa, and Inaba have been prepared, but work on *V. cholerae* O139 has been hindered by the challenging synthesis of the critical hexasaccharide (Figure 11) [80]. From the four oral-inactivated or non-live cholera vaccines available, only two contain the *V. cholerae* serogroup O139: (1) ShanChol (Shantha Biotechnics Limited, India) [81] and (2) Euvichol (Eubiologics, South Korea) [82]; however, these cholera vaccines are World Health Organization prequalified and not available in the United States [83].

The first chemical synthesis of the complete protective ready-for-conjugation antigen of *V. cholerae* O139 [80] was improved from a small-scale chemical synthesis into a less demanding experimental method. A linker-equipped synthesis of the O-specific polysaccharide of *V. cholerae* O139 allowed for conjugation to a carrier protein, BSA (Figure 11) [84]. Glycoclusters displaying synthetic oligosaccharides of the O-specific polysaccharide of *V. cholerae* O1 ST Inaba were conjugated to BSA. The immunoreactivity of the glycoclusters was studied using plasma of patients recovering from cholera. The synthetic glycoclusters showed similar immunoreactivity compared with conventional oligosaccharide conjugates from wild-type bacteria [85].

### *Burkholderia pseudomallei* and *Burkholderia mallei*

*Burkholderia pseudomallei* (*B. pseudomallei)* is a bacterium causing melioidosis leading to death in up to 50% of patients. *Burkholderia mallei* (*B. mallei)* is a bacterium that causes glanders in solipeds; however, it may be transmitted into humans leading to fatal infections if left untreated. Currently, there are no clinically approved prophylactic vaccines on the market for either of these pathogens [86].

**Figure 12.**
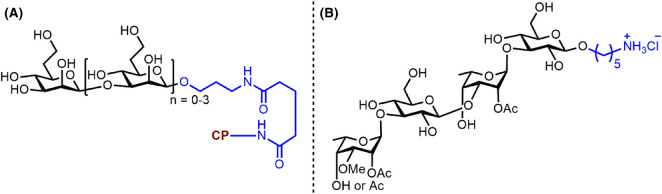
Synthetic capsular polysaccharides from Burkholderia. (**A**) Homologated polymannosides confer T-cell-dependent immunity against *B. pseudomallei* and *B. mallei* [89]. (**B**) Synthetic lipopolysaccharide O-polysaccharide of *Burkoholderia* [90].

A synthetic hexasaccharide fragment from the CPS of *B. pseudomallei* was coupled to the nontoxic H_C_ domain of TT, showing immunologically relevant and protective properties in mice [87]. Seven minimal epitopes from *B. pseudomallei* and *B. mallei* lipopolysaccharide O-antigens were synthesized. Immunization of mice using the two terminal disaccharides that comprise the lipopolysaccharide O-antigens’ epitopes from *B. pseudomallei* and *B. mallei* conjugated to the CRM_197_ carrier protein showed high-titer IgG responses against one of the disaccharides [88]. Synthetic fragments of the common and major capsular polysaccharide, type I O-PS from *B. pseudomallei* and *B. mallei* conjugated to CRM_197_ carrier protein elicited high titers of IgG antibodies and a robust T-cell-dependent immune response in mice [89] (Figure 12A). Tetrasaccharide structures of the native O-polysaccharide of LPS from *Burkoholderia* sp. were found to be strongly reactive in sera from meliodosis patients (Figure 12B) [90].

## Conclusions and outlook

Due to their proven success, antibacterial semisynthetic and fully synthetic vaccines will remain an important area of research. Since the first marketed carbohydrate-based vaccine in 1947 and the first semisynthetic carbohydrate-based vaccine commercialized in 2004, the field has advanced steadily. Just the last five years covered in this mini-review have seen impressive developments.

Recent advances have offered vaccine candidates composed of adjuvants on a sole (macro)molecule triggering T and B cells concurrently, regardless of a carrier protein. Alongside novel synthetic tools, as MPLA incorporated liposomal, carbohydrate-based particles, and additional non-protein T cell-activating carriers, the field of antibacterial carbohydrate vaccines holds great promise.

To produce a commercial synthetic vaccine, limiting the number of requisite synthetic steps while still achieving the desired, specific immune response is of fundamental importance. Synthetic chemistry is cumbersome and time-consuming, often providing the greatest bottleneck in pharmaceuticals. Despite the inherent possibility to expedite production and enhance the purity of the overall vaccine formulation by depending on synthetic chemistry, a minimalistic strategy — hopefully facilitated through improvements in automated glycan assembly — is paramount to availing the first licensed fully synthetic vaccine. At this time, targeting *S. pneumoniae* may hold the most promise toward achieving a synthetic vaccine, but many infections from other bacteria, such as *N. meningitidis*, could soon be prevented.

The COVID-19 pandemic highlights the urgent need for efficient vaccines, and the increased interest in vaccines of all varieties among the general population does offer excitement. We note that much like with the SARS-CoV-2 vaccines, providing availability to remote areas while maintaining affordability remains a challenge.

## Perspectives

- *Importance to the field:* Although proven highly effective against human pathogenic bacteria for more than three decades, purified capsular polysaccharides for glycoconjugate vaccine production still pose many challenges. Semi- and fully synthetic glycoconjugate vaccines are steadily emerging as elegant solutions that bypass the need for native carbohydrates while providing highly effective, long-lasting immune protection.
- *Summary of current thinking:* Although different exceptions were demonstrated, the ability to induce an adequate, long-lasting T cell-dependent immune protection against bacterial capsular polysaccharides is likely a combination of potent adjuvants, immunogenic carrier, and properly presented carbohydrate antigens by the MHC-II complex.
- *Future directions:* The greatest bottleneck in the design of semi- and fully synthetic vaccines may always remain the synthesis itself. Facilitating access to the active components of the vaccines in high purity at scale is an ongoing, longstanding interest in the field of vaccine design. Thus far, automated solid-phase methods have shown the most promise, with automated glycan assembly having an appreciable lead in this regard. Synthetic methodological advancements that are amenable to automated glycan assembly are certainly the way forward.

## Open Access Statement

Open access for this article was enabled by the participation of Max Planck Digital Library in an all-inclusive Read & Publish pilot with Portland Press and the Biochemical Society under a transformative agreement with Max Planck Digital Library.

## Abbreviations

BSA: bovine serum albumin
CPS: capsular polysaccharide
D-AAT: 2-acetamido-4-amino-2,4,6-trideoxy-D-galactose
DT: diphtheria toxoid
GAS: Group A *Streptococcus*
GBS: Group B *Streptococci*
MHC: major histocompatibility complex
MPLA: monophosphoryl lipid A
OMPC: outer membrane protein complex
ST: serotypes
TT: tetanus toxoid
VLP: virus-like particle
